# A Deep Siamese Convolution Neural Network for Multi-Class Classification of Alzheimer Disease

**DOI:** 10.3390/brainsci10020084

**Published:** 2020-02-05

**Authors:** Atif Mehmood, Muazzam Maqsood, Muzaffar Bashir, Yang Shuyuan

**Affiliations:** 1 School of Artificial Intelligence, Xidian University, No. 2 South Taibai Road, Xian 710071, China; atifedu151@yahoo.com; 2 Department of Computer Science, COMSATS University Islamabad, Attock Campus, Attock 43600, Pakistan; muazzam.maqsood@cuiatk.edu.pk; 3 Department of Physics, University of the Punjab, Lahore 54590, Pakistan

**Keywords:** Alzheimer’s disease, dementia, convolutional neural network, classification, deep learning, batch normalization

## Abstract

Alzheimer’s disease (AD) may cause damage to the memory cells permanently, which results in the form of dementia. The diagnosis of Alzheimer’s disease at an early stage is a problematic task for researchers. For this, machine learning and deep convolutional neural network (CNN) based approaches are readily available to solve various problems related to brain image data analysis. In clinical research, magnetic resonance imaging (MRI) is used to diagnose AD. For accurate classification of dementia stages, we need highly discriminative features obtained from MRI images. Recently advanced deep CNN-based models successfully proved their accuracy. However, due to a smaller number of image samples available in the datasets, there exist problems of over-fitting hindering the performance of deep learning approaches. In this research, we developed a Siamese convolutional neural network (SCNN) model inspired by VGG-16 (also called Oxford Net) to classify dementia stages. In our approach, we extend the insufficient and imbalanced data by using augmentation approaches. Experiments are performed on a publicly available dataset open access series of imaging studies (OASIS), by using the proposed approach, an excellent test accuracy of 99.05% is achieved for the classification of dementia stages. We compared our model with the state-of-the-art models and discovered that the proposed model outperformed the state-of-the-art models in terms of performance, efficiency, and accuracy.

## 1. Introduction

Alzheimer’s disease (AD or Alzheimer’s) causes the loss of tissues and death of nerve cells throughout the brain, resulting in memory loss of humans and imposing a bad impact on the performance of routine life tasks such as writing, speaking, and reading. Sometimes AD Patients may have problems in the identification of their family members. Mild cognitive stage patients behave very aggressively, but patients in the last stage of AD suffer from heart failure and respiratory system dysfunctionality leading to death [1]. Early and accurate diagnosis of AD is not possible due to the improper medication that has been specified [2]. However, the early-stage diagnosis of Alzheimer’s and treatment can improve the patient’s life [3]. All the indicators of AD usually grow slowly but affect severely with the passage of time, when the disorder of the human brain starts [4]. Every year a large number of people suffer from this disease. As per an estimation, one out of 85 persons would be suffering from AD in the world till 2050 [5]. The global deterioration scale (GDS) is commonly used for dementia scaling. This scale further divides AD into seven stages, which depend on the value of cognitive decline. In GDS, stage 4 is considered as early dementia; however, stage 5 and stage 6 are treated as middle dementia. On the other hand, the clinical dementia rating (CDR) scale is also used in dementia research because it is easy for communication between the families and medical professionals [6]. CDR scores assigned to test six different parameters such as problem-solving, orientation, hobbies, memory, and judgment [7].

In AD patients, the cerebral cortex and hippocampus sizes shrink in the brain, but the size of the ventricles increases in the brain. Spatial memory and episodic memory are part of the brain that is damaged when the hippocampus size is reduced [8]. It also provides connectivity between the brain and body. Hippocampus reduction causes cell loss and impairment of the synapses and neuron ends [9]. Due to the uncertainty between neurons, communication defects in the short-term memory, planning, and judgment have been observed [10]. Researchers have established many Computer-Aided Diagnosis Systems (CADS) for the accurate detection and classification of the extracted features related to AD [11]. To process the extracted features, more effort and time by human experts is required otherwise.

Recently, the researchers are developing deep learning models/techniques to extract the features directly from medical images [12]. Deep learning models have achieved major conquest in medical images such as CT, MRI, X-ray, microscopy, and mammography [13]. These models or methods mainly focused on binary classification, which shows only that whether the patient is suffering from AD or not [14]. However, proper diagnosis of the patient requires the classification of different dementia stages. MCI stage is a highly defective stage as compared to AD because 10% to 16% of patients convert MCI sharply to AD per year [15]. MCI stage is highly variable for patients to stabilize or reverts into the healthy stage [16]. However, for prediction, the conversion from MCI to AD important biomarkers are Magnetic Resonance images (MRI) because they are less expensive as compared to positron emission tomography (PET) and cerebral spinal fluid (CSF). MRI based images contain multi-modal information regarding the function and structure of the brain, which is suitable for clinical purposes.

A large number of datasets produced massive progress in object detection and image classification because most datasets are labeled. A common example is an ImageNet database, which has millions of better images for model learning. Convolutional neural networks (CNNs) gave a robust performance on large image datasets [17]. In deep learning, CNN is widely known for its ability to perform high accuracy in terms of medical image classification. There are different models that are utilizing CNN for AD scan categorization. However, the most important advantage of CNNs as compared to conventional machine learning techniques is that CNNs do not require manual feature extraction because CNNs are capable of extracting the effective features automatically and then categorizing the stages of AD [18]. However, existing machine learning and deep learning models are trained from scratch but have some limitations such as, (1) to train the deep learning model on huge number of images requires massive computational resources; (2) for the proper training of the model, we need magnificent amount of standard training datasets, which is the biggest problem for medical imaging where standard data can be expensive and ethically privacy issues arises; (3) during training the model on medical imaging requires more attentive and tedious tuning of a number of parameters, which cause overfitting problems and affect the overall performance of the model.

Our current research towards the development of the Siamese convolutional neural network (SCNN) inspired by the VGG16 architecture [19] is implemented for the improved diagnosis and classification of multiple stages of Alzheimer’s from no dementia to moderate AD. The model is completely identical but joins two modified VGG16 parallel layers, “Siamese”. We insert a concatenation that joins every single layer. The key objective of the proposed technique is to reduce the dependency on large datasets. We acquired the 3-D views of the human brain dataset from the OASIS repository and achieved better performance as compared to the state-of-the-art performances on small MRI images. In this study, our key contributions are given below:

- We formed an SCNN model for the multi-class classification of Alzheimer’s disease.
- We presented an efficient model to overcome the data shortcoming complications for an imbalanced dataset.
- We developed a regularized model that learns from the small dataset and still demonstrates superior performance for Alzheimer’s disease diagnosis.

Recently several researchers have developed techniques for AD diagnosis. The techniques are categorized based on machine learning and deep learning models. These techniques are briefly discussed in the following section.

### 1.1. Machine Learning-Based Technique

Several machine learning-based models have been proposed to extract the features and perform multiple operations on AD MRI images [20]. Kloppel et al. [21] developed a dimensional reduction model to detect AD patients by using a linear support vector machine on T1-weighted MRI images. Gray et al. [22] used a random forest classifier to develop a multimodal classification for AD classification on positron emission tomography (PET) and MRI data. Morra et al. [23] introduced different models’ comparison to detect AD on MRI scans such as SVM and hierarchical AdaBoost models. Neff et al. [24] developed an algorithm for feature extraction and reduction by using downsized kernel principal component analysis (DKPCA) and support vector machine (SVM) for AD MRI images. They tested the model on the OASIS datasets and obtained 92.5% accuracy using a multi-support vector machine (MSVM) kernel. Wang et al. [25] used wavelet entropy and biography-based optimizers to extract the features in MRI data and classified them. They obtained 100% accuracy by applying a six-fold CV model on 64 brain images. Ding et al. [26] have improved the feature extraction and feature selection accuracy on AD and NC patient’s datasets. They used gray level occurrence matrix and voxel based morphometric (VBM analysis for feature extraction and SVM for classification purposes. Performance checked on the Alzheimer’s disease Neuroimaging Initiative (ADNI) dataset showed an accuracy of 92.86%. Dashan et al. [27] proposed systems for feature extraction and reduction on T2-weighted MRI images produced by Harvard medical school. Two classifiers are being tested on the same dataset and obtained an accuracy between 97% to 98%. Hinrich et al. [28] obtained samples from the ADNI database and applied the proposed technique on the multi-classification of Alzheimer’s. The overall accuracy obtained was 79.8% for all stages. Yue et al. [29] also developed a voxel-based hierarchical feature extraction technique that finds the correlation between subjects. In the second step, feature vectors were used for processing the feature and placed into the classifier to check the effectiveness. Ahmed et al. [30] designed a simpler CNN model using the patch-based classifier to diagnosis the AD multi-stages. The model reduced the computational cost and produced a great improvement in accuracy. They generated the patches on a three-view of MRI image and obtained an overall 90.05% accuracy. However, utilizing machine learning models with the hand-crafted features, most studies showed accuracy that depended on how well the feature was defined. For this purpose, the domain experts are required to achieve maximum performance. For such a limitation, one of the solutions is deep learning as it is familiar to capture arbitrary features automatically and then achieve relatively high accuracy [31].

### 1.2. Deep Learning-Based Technique

Several deep learning-based models have been proposed to extract the features directly on input data and perform multiple operations on MRI images [32]. These models based on multiple layers and hierarchical structure, which rapidly increased the ability of feature representation on different datasets. Liu et al. [33] have adopted a zero-masking strategy (ZMS) to develop a model that has the ability to prevent the maximum data loss of the MRI image data. Gupta et al. [34] introduced a sparse auto-encoder based model for the classification of three AD stages. The respective accuracy obtained on the ADNI dataset for multi-classification is 95%. Dou et al. [35] presented the improved performance model on 3D CNN and 2D CNN approaches. They used 3D CNN model to detect cerebral micro-bleeds. They applied the extensive experiment to validate the purposed model and obtained the sensitivity result of 93.16%. Suk et al. [36] proposed another technique to classify AD and MCI converter stages by using an auto-encoder network. They obtained an accuracy rate of 95.9% over these MCI stages. Hong et al. [37] predicted Alzheimer’s disease using long short-term memory (LSTM) because it was able to connect the patient’s previous information to the current task. They process the time series data in three layers, such as pre-fully connected, cells, and post-fully connected layers. The accuracy is still limited because to get traditional features from temporal information due to the lack of data. They obtained the 82.05% overall performance for multi-classification of AD patients. The authors obtained at best 98.78% multiclass—classification accuracy on the OASIS data using ResNet50 and gradient boosted machine [38]. In [39], the researchers proposed a deep learning model using inceptionV3 architecture for the early diagnosis of Alzheimer’s disease to test on the ADNI dataset. They analyzed the accuracy rate on receiving operating characteristic (ROC) is 95% and sensitivity 100%. One such recent method developed [40] for Alzheimer’s diagnosis and multi-classification from MRI images with the help of intelligent data selection. They used the popular CNN architecture VGG on the ADNI database. They also deployed the transfer learning and showed very high classification performance, such as for AD vs. NC 99.36%, MCI vs. NC 99.04%, and 99.20% overall accuracy for multi-classification. In [41] researcher proposed CNN based model and acquired 94.54% classification accuracy for early mild cognitive impairment (EMCI) and late mild cognitive impairment (LMCI). The exiting studies using deep learning for medical images and text classification, the CNN provides improvement in results by automatically learning the features on the given task. However, if we compare RNN, CNN has a smaller number of parameters, so CNN is more suitable for a small number of datasets [42].

## 2. Materials and Methods

**Figure 1:**
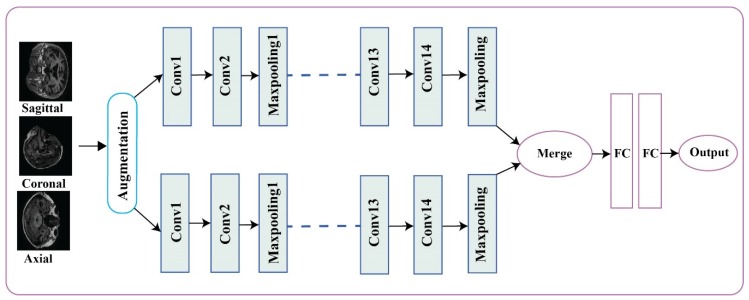
Proposed siamese convolutional neural network (SCNN) model for the Classification of Alzheimer’s Stages.

In the proposed approach, the algorithm depends on three steps. The first step is data preprocessing and augmentation, the second stage is feature extraction from input images, and the third step is the classification of dementia classes. We developed a CNN-based approach inspired by VGG16 for the classification of dementia stages. We modified the VGG16 and inserted one extra Conv layer in the model which was effective to grasp maximum features on a small dataset [43]. In the algorithm, two modified VGG16 layers were working parallel with 14 Conv layers, five max-pooling, three batch normalization, and three Gaussian noise. The reason for the model is the parallel work to extract the more important features. Consecutive parallel layers improved the classification accuracy [44]. Table 1 shows the complete details about the pool size and the number of kernels in our proposed model. The experimental dataset is based on the clinical dementia rating (CDR) score. The work-flow of the proposed model is shown in Figure 1.

### 2.1. Data Selection

In our research work, we used OASIS open-access dataset. These datasets investigate during preparation by Daniel S. Marcus from Neuroimaging Informatics Analysis Center (NIAC) at Washington University School of medicine. We have 382 images obtained from the OASIS database. We create four classes (Table 2) based on CDR score such as CDR-0 (No Dementia), CDR-0.5 (Very Mild Dementia), CDR-1 (Mild-Dementia), and CDR-2 (Moderate AD). Available Alzheimer’s disease patients have aged in the range of 20 to 88 years. We apply the augmentation approach to create balanced data to improve the model learning rate. The data preprocessing is the major part to extract efficient and accurate results for those algorithms based on the CNN model. The OASIS [45] dataset image size is 256 × 256 but the proposed CNN model requires an image size of 224 × 224. For this purpose, image dimension setting scaling is applied to the OASIS dataset.

### 2.2. Image Preprocessing

For the proposed model, the training and testing on medical images go through the preprocessing steps. MRI images during the process of their forming endure deterioration, such as low variation due to bad brightness produced by the visual devices. To overcome this issue for the improvement of MRI scans, image enhancement approaches were applied for the upgrade of the distribution of pixels over an extensive range of intensities, linear contrast stretching was applied on the images. During the image acquisition process, some undesirable information was added to the image due to nonlinear light intensity conceded as noise. Specifically, non-linear light intensity affects the overall performance-accuracy of the image processing [46]. Due to the improper setting of the lens slit of the scanning devices, non-linear light was mostly introduced and the uneven distribution of light was normalized by image enhancement techniques. The dynamic range of light intensity was increased by using contrast stretching because the output images after this process were the ones having improved contrast and appropriate light distribution. Images in the OASIS repository to get better performance on the latter stages were enhanced using the linear contrast stretching. MRI images were obtained from the public OASIS repository and were segmented by extracting the differing intensities of the white matter (WM), gray matter (GM), and cerebrospinal fluid (CSF) by using the K-mean clustering. Segmented images were resized to 224 × 224 as per model requirements.

### 2.3. Data Augmentation

In neuroimaging, a large number of scans related to AD patient’s availability are a major issue because few hundreds of image samples are available. It is a common thing for a deep learning model to provide more effective results on more data. In medical research, due to privacy concerns, the access to large data is a big problem [47], especially, the classification of cancer and AD are problematic due to lack of availability of data. The small imbalanced dataset creates overfitting problems during training of the model which affects the model efficiency. To overcome this issue, we need more data to enhance the effective accuracy in our proposed model. We used the augmentation technique to create 10 more images on each available MRI image [48]. In Table 3, data augmentation is described for the parameters used for augmentation.

### 2.4. Convolutional Neural Networks

In the core, the convolutional neural network number of layers extracts local feature on large dimensional data. Each layer consists of distinct nodes with learnable bias and weights; in the convolutional layer’s connection, weights are shared and called a convolution kernel. All the operation results are decided by a different number of activation functions. For the dimensionality reduction and moderation, the sample data used the pooling layer. In CNN, the output of the previous layer is convolved with a learnable kernel and weight-sharing plays a key role in training to reduce the number of weights. The general formula for a convolutional layer is given by:

(1) $$\mathrm{Height}\;=\;\frac{\mathrm{Image}\;\mathrm{hegiht}\;-\;\mathrm{kernel}\;\mathrm{height}\;+\;2\left(\mathrm{padding}\right)\;}{\mathrm{strides}}\;+\;1,$$

(2) $$\mathrm{Width}\;=\;\frac{\mathrm{Image}\;\mathrm{width}\;-\;\mathrm{kernel}\;\mathrm{width}\;+\;2\left(\mathrm{padding}\right)\;}{\mathrm{strides}}\;+\;1,$$

(3) $$C\;=\;\frac{W\;-\;K\;+\;2P}{S}\;+\;1\;,$$

The convolution layer W denotes the image height and width, K represents the filter size, P is the padding, and S refers to the strides. The pooling layer inserted between convolution layers to reduce the computational complexity by a down sampling operation, mostly the max-pooling layer is commonly used. The output of the feature map by convolutional layer further divide into small regions, and each region described the value of the region. CNN is commonly based on the number of pairs of convolutional and pooling layers, successfully connected and finally, softmax to produce the final output labels. In CNN’s training, backpropagation [49] is used in order to reduce the cost function and each layer’s weights are iteratively updated. In our model, we define a kernel initializer as “random uniform” and bias initializer “zeros”. We used the sequential to create a layer by layer model with Rectified Linear Unit (ReLU) activation function. VGG16 refers to the layers that have weights, more detail is shown in Table 1.

### 2.5. Improved Learning Rate and Regularization

Training the CNN model is very difficult because the input of each layer changed when we change the parameters of the previous layer. On the other hand, activation functions such as sigmoid and ReLU lose their gradient rapidly, which creates a problem for learning in deep neural networks. Due to this issue, the learning rate of the model slows down gradually. To overcome this issue, we used in our proposed model batch normalization [50]. Batch normalization produced a high learning rate on the model and also reduced the parameter initialization [51]. During data reduction, the internal covariant is shifted and the mean and variance values are fixed in the input layers.

(4) $$y_{i}\;={\;\mathrm{BN}}_{\gamma ,\beta }\left(x_{i}\right),$$

(5) $$\mu_{b}\;=\;\frac{1}{n}\sum_{i=1}^{n}x_{i},$$

(6) $$\sigma_{b}^{2}\;=\;\frac{1}{n}\sum_{i=1}^{n}{\left(x_{i}-\mu_{b}\right)}^{2}\;,$$

(7) $$\bar{x_{i}}\;=\;\frac{x_{i}-\mu_{b}}{\sqrt{\sigma_{b}^{2}+\in }},$$

(8) $$y_{i}\;=\;\gamma *\bar{x_{i}}+\beta ,$$

where n represent the number of batches and $\mu ,\sigma^{2}$ mean and variance, $x_{i}$ represent each row. By using Equations (4)–(6), the mean and variance of each activation across a mini-batch are calculated. In Equation (7), there are two hyper-parameters, γ and β, which produced the learnable scale parameters for each input dimension. On the other hand, we inserted Gaussian noise [52] to improve the robustness and regularization of our model. Gaussian noise produces very effective results during the training of the deep model and also helps to decrease the training loss.

### 2.6. Alzheimer’s Disease Detection and Classification Architecture

In our model, 14 convolutional layers with the ReLU activation function were working in parallel with three batch normalization, three Gaussian noise, and five max-pooling layers with two stride sizes. We used the Adamax optimizer with 0.002 learning rate, and categorical cross-entropy as a loss function was used in our model, as shown in Table 1.

## 3. Results

**Figure 2:**
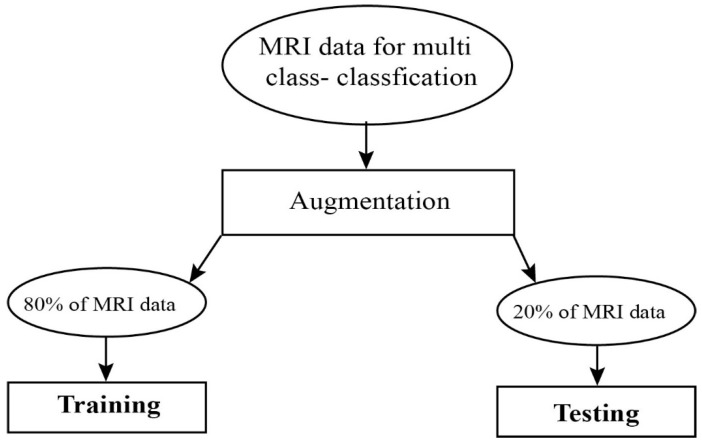
Proposed model data flow chat.

We used Keras library for the implementation of our model on Z840 workstation Intel Xeon (R) CPU E5-2630v3 @2.40GHz*32 with 64 GB memory. To validate the effectiveness of the proposed CNN based approach with an extra convolutional layer, which is inspired by VGG16 architecture, was used to classify Alzheimer’s disease. We extracted the feature from the 3820 data samples after preprocessing. We divided the dataset for training 80% and testing 20% which belonged to four classes, more detail is shown in Figure 2. We used full test data as validation data and validated the model, so the final epoch result of the validation accuracy could be said, as test accuracy or validation accuracy. To stop the overfitting, we used early stopping. The classification results obtained by the proposed model were evaluated using the different evaluation metrics [6], and we obtained 99.05% test accuracy. We used the Monte Carlo method to check the significance of the classification results under optimal parameters. We performed the analysis on the different number of epochs (5, 10, 15, and 20) with varying classification results such as (0.97, 0.98, 099, and 0.98), we noticed that the average performance results were achieved on 15 epochs and confusion matrix of our model is shown in Table 4.

**Figure 3:**
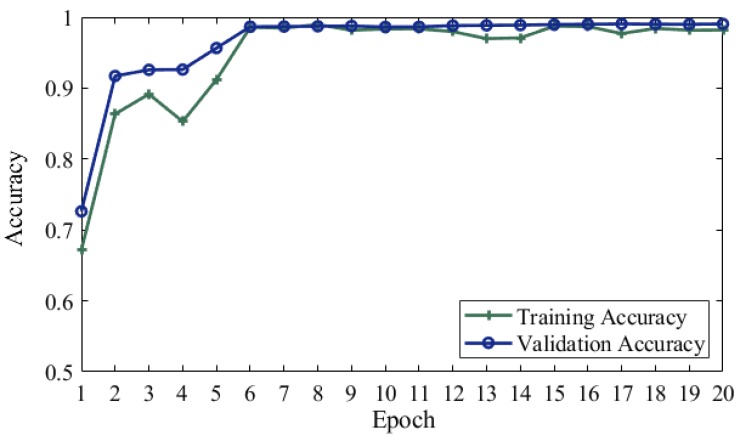
Proposed model training and validation accuracy.

**Figure 4:**
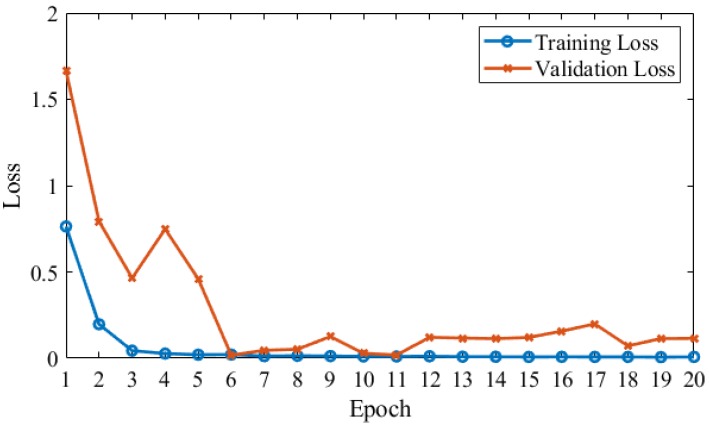
Proposed model training and validation loss.

In our proposed approach, we used CNN based parameters for training the model parallel on the same input and extract the features on the images to find the desired output. Figure 3 shows that training and validation accuracy with data augmentation and Gaussian noise on MRI data. Our proposed model achieved a 99.05% test accuracy. Figure 4 shows a blue line for training loss and an orange line for validation loss. In proposed model validation data attained 67.13% accuracy on first epoch, 86.35% on second, 89.16% on third, 85.29% on fourth, 91.19% on fifth epoch and last epoch attained 98.19%. Validation epochs show the accuracy decrease 1.02% on 13 epoch size and 1% decrease for epoch size 17.

**Figure 5:**
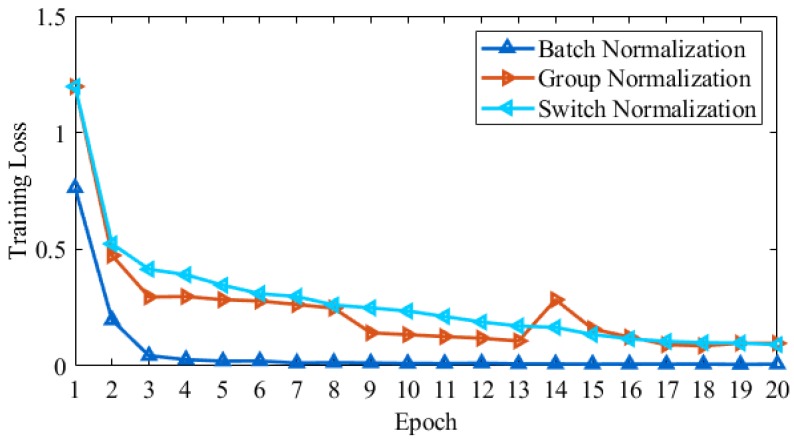
Training loss results with three normalization techniques on the AD dataset.

Figure 5 shows the results for three normalizations, such as Batch Normalization (BN), Group Normalization (GN), and Switch Normalization (SN) used in the proposed model. For Alzheimer’s disease multi-class classification, we compared the training loss achieved from three normalization types on a different number of epochs. Switch normalization produced the maximum loss on each epoch. However, group normalization performance increased to maximum on epoch size 16, but Batch normalization produced the best results in terms of training loss during the whole model training process.

**Figure 6:**
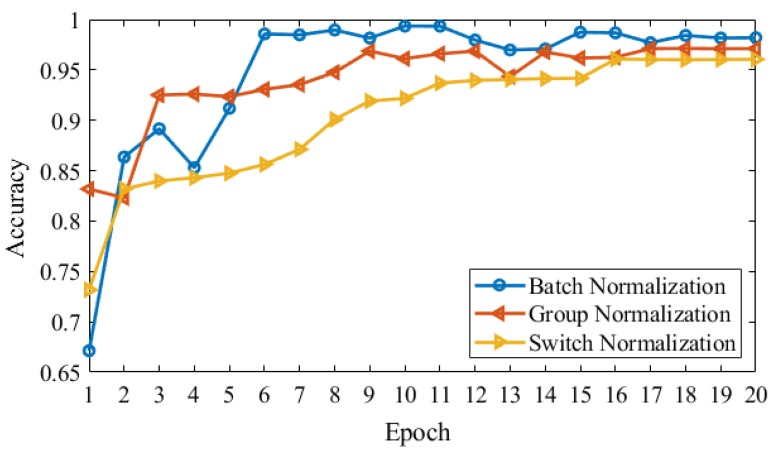
Validation accuracy with three normalization techniques applied to the AD dataset.

In Figure 6, we show the validation accuracy using three types of normalizations in our model. In a multi-classification of Alzheimer’s disease stages, group normalization produced 80% on epoch 4 and epoch 5 as compare to other normalization. Switch normalization produced a maximum of 96.05% in terms of validation accuracy. On the other hand, batch normalization attained high performance on validation accuracy 98.17% on 19 epoch and 98.19% on 20 epoch.

## 4. Discussion

In this research work, two pipelines working parallel and joint on end are used to predict the multi-class classification results on dementia stages. In the four-way classification of no dementia (ND), very mild dementia (VMD), mild dementia (MD), and moderate AD (MAD) from the OASIS dataset, and overall accuracy obtained is 99.05%. We compared our results based on the proposed SCNN model that applies deep learning on OASIS and ADNI datasets with five state-of-the-art methods. We discovered that the accuracy of our technique with four stages data significantly exceeded state of the art. Especially for mild dementia and moderate AD classification problems, we obtained a 5% improvement over 3D CNN. We can see in Table 5 the accuracy of approaches is above 90% generally. Our method produced more improvements in the results to train the model parallel, to reduce the overfitting and regularize on small datasets. Although an increased number of samples would improve the accuracy of the model to obtain the annotated data for medical images is very difficult. Using data augmentation, we are able to train our model because augmentation approaches produced effective results for clinical applications. Further, we used batch normalization and distinguished with Gaussian noise. Figure 6 shows the comparison, where we utilize three types of normalizations in our model to check the validation accuracy, such as Batch normalization, group normalization, and switch normalization. However, batch normalization outperformed the rest of the others.

In Islam et al. [53], five approaches were introduced for multi-classification; as reported in the paper, strong gradient flow in the training data increased the performance by using inception-v4 and ResNet. On the other hand, the remaining three approaches generate poor performance. The author used ResNet with MRI scans on OASIS and produced 93.18% accuracy. Hosseini et al. [54] used the 3D-DSA-CNN technique and generated the 97.60% accuracy value for each group. Besides, researchers in [55] introduced the Sobolev gradient-based stochastic optimizers used in 3D-CNN to diagnose the AD and obtained the 98.01% accuracy. Another study by Khan et al. [40] solved the issue with transfer learning and optimized the VGG architecture for the multi-classification of AD. They introduced the new method for layer-wise tuning, to find out the more informative slices in the data they applied the image entropy. Feature selection without overfitting is a big challenge to improve the model classification accuracy. Bijen et al. [14] introduced the fine-tuned pre-trained CNN for feature extraction purposes. They applied the simpler machine learning model and obtained the time-efficient and desirable results in the sense of classification accuracy on the whole-brain model.

Finally, it can be seen that for the multi-classification problem our proposed model achieves state of the art results when we compared it with the existing models. We also provide a deeper analysis of our proposed technique to extract useful information from MRI slices. The work had a lot of limitations which affect accuracy result. First, there is a smaller number of annotated data. Second, preprocessing steps such as skull stripping, segmentation and normalization have convoluted parameters, which are the big problems to deal with a number of parameters correctly.

## 5. Conclusion

In this research article, we proposed a deep learning model to predict the multi-class classification of stages of Alzheimer’s disease. Our proposed SCNN model is inspired by VGG-16. We approve our model with detail experiments on the OASIS dataset, where MRI image belongs to four categories such as no dementia (ND), very mild dementia (VMD), mild dementia (MD), and moderate AD (MAD) is used to obtain the highest accuracy of our proposed approach. We also investigate the reduction of overfitting and regularization of the model effect on our application performance. For this purpose, we used three types of normalizations and Gaussian noise. Finally, we compare our proposed technique to the existing five state-of-the-art approaches, where our proposed model significantly performed better than the others. We can see our proposed approach providing a 3% to 6% improvement for multi-class classification as compared to the state-of-the-art techniques.

In the future, we will examine whether the same model can be employed on the other computer-aided diagnostic problems. We will also investigate further improvement by an intelligent splitting of training data for classification.

## Figures and Tables

**Table 1:** Feature extraction and classification layers in the proposed SCNN model.

Layer No.	Layer Name	Kernel Size	Pool Size	No. of Filters
**1**	Conv1 + ReLU	3		64
	Batch Normalization			
**2**	Conv2 + ReLU	3		64
	Maxpooling1		2	
**3**	Conv3 + ReLU	3		128
	Gaussian Noise			
	Batch Normalization			
**4**	Conv4 + ReLU	3		128
	Maxpooling2		2	
**5**	Conv5 + ReLU	3		256
	Batch Normalization			
**6**	Conv6 + ReLU	3		256
**7**	Conv7 + ReLU	3		256
	Gaussian Noise			
**8**	Conv8 + ReLU	3		256
	Maxpooling3		2	
**9**	Conv9 + ReLU	3		512
**10**	Conv10 + ReLU	3		512
**11**	Conv11 + ReLU	3		512
	Maxpooling4		2	
**12**	Conv12 + ReLU	3		512
	Gaussian Noise			
**13**	Conv13 + ReLU	3		512
**14**	Conv14 + ReLU	3		512
	Maxpooling5		2	
**15**	Flatten1			
**16**	Flatten2			
**17**	Concatenate			
**18**	FC1 + ReLU 4096			
**19**	FC2 + ReLU 4096			
**20**	Softmax			

**Table 2:** Summary of the global clinical dementia rating (CDR).

Clinical Dementia Rate (RATE)	No. of Samples
CDR-0 (No Dementia)	167
CDR-0.5 (Very Mild Dementia)	87
CDR-1 (Mild-Dementia)	105
CDR-2 (Moderate AD)	23

**Table 3:** Data augmentation.

Rotation Range	10 Degree
Width shift range	0.1 Degree
Height shift range	0.1 Degree
Shear range	0.15 Degree
Zoom range	0.5, 1.5
Channel shift range	150.0

**Table 4:** Proposed model confusion matrix on the OASIS data.

Actual Class		Predicted	Class	
	ND	VMD	MD	MAD
No Dementia (ND)	334	0	0	0
Very Mild Dementia (VMD)	0	170	4	0
Mild Dementia (MD)	0	3	207	0
Moderate AD (MAD)	0	0	0	46

**Table 5:** Comparison of accuracy values with state-of-the-art techniques applied to diagnose Alzheimer’s disease stages.

Paper	Method	Dataset	Accuracy
Islam et al. [53]	ResNet, CNN	OASIS (MRI)	93.18%
Hosseini et al. [54]	3D-DSA-CNN	ADNI (MRI)	97.60%
Evign et al. [55]	3D-CNN	ADNI (MRI)	98.01%
Farooq et al. [56]	GoogLeNet	OASIS (MRI)	98.88%
Khan et al. [40]	VGG	ADNI (MRI)	99.36%
Proposed Method	SCNN	OASIS (MRI)	99.05%

## References

[1] Beheshti I., Demirel H., Neuroimaging D. (2016). Feature-ranking-based Alzheimer’s disease classification from structural MRI. Magn. Reson. Imaging.

[2] Alzheimer’s Association (2019). 2019 Alzheimer’s disease facts and figures. Alzheimer’s Dement..

[3] Afzal S., Maqsood M., Nazir F., Khan U., Song O. (2019). A data augmentation-based framework to handle class imbalance problem for Alzheimer’s stage detection. IEEE Access.

[4] Picón E., Rabadán O.J., Seoane C.L., Magdaleno M.C., Mallo S.C., Vietes A.N., Pereiro A.X., Facal D. (2019). Does empirically derived classification of individuals with subjective cognitive complaints predict dementia?. Brain Sci..

[5] Brookmeyer R., Johnson E., Ziegler-graham K., Arrighi H.M. (2007). Forecasting the global burden of Alzheimer’s disease. Alzheimer’s Dementia.

[6] Maqsood M. (2019). Transfer learning assisted classification and detection of Alzheimer’s disease stages using 3D. Sensors.

[7] Bryant S.E.O., Waring S.C., Cullum C.M., Hall J., Lacritz L. (2015). Staging dementia using clinical dementia rating scale sum of boxes scores. Arch. Neurol..

[8] Alirezaie J., Jernigan M.E., Nahmias C. (1998). Automatic segmentation of cerebral MR images using artificial neural networks. IEEE Trans. Nucl. Sci..

[9] Xie Q., Zhao W., Ou G., Xue W. (2019). An overview of experimental and clinical spinal cord findings in Alzheimer’s disease. Brain Sci..

[10] Sarraf S., Tofighi G., Neuroimaging D. (2016). DeepAD: Alzheimer’s disease classification via deep convolutional neural networks using MRI and fMRI. BioRxiv.

[11] Hosseini-Asl E., Keynton R., El-Baz A. Alzheimer’s disease diagnostics by adaptation of 3D convolutional network. Proceedings of the International Conference on Image Processing.

[12] Litjens G., Kooi T., Bejnordi B.E., Arindra A., Setio A., Ciompi F., Ghafoorian M., Van Der Laak J.A.W.M., Van Ginneken B., Clara I.S. (2017). A survey on deep learning in medical image analysis. Medical Image Analysis.

[13] Hosny A., Parmar C., Quackenbush J., Schwartz L.H., Aerts H.J.W.L. (2018). Artificial intelligence in radiology. Nat. Rev. Cancer.

[14] Khagi B., Kwon G.R., Lama R. (2019). Comparative analysis of Alzheimer’s disease classification by CDR level using CNN, feature selection, and machine-learning techniques. Int. J. Imaging Syst. Technol..

[15] Huang C., Slovin P.N., Nielsen R.B., Skimming J.W. (2002). Diprivan attenuates the cytotoxicity of nitric oxide in cultured human bronchial epithelial cells. Intensive Care Med..

[16] Ritchie K., Ritchie C.W. (2012). Mild cognitive impairment (MCI) twenty years on. Int. Psychogeriatrics.

[17] Fei-Fei L., Deng J., Li K. (2010). ImageNet: Constructing a large-scale image database. J. Vis..

[18] Baron J.C., Chételat G., Desgranges B., Perchey G., Landeau B., De La Sayette V., Eustache F. (2001). In vivo mapping of gray matter loss with voxel-based morphometry in mild Alzheimer’s disease. Neuroimage.

[19] Arge F.O.R.L., Mage C.I. (2015). V d c n l -s i r.

[20] Plant C., Teipel S.J., Oswald A., Böhm C., Meindl T., Mourao-Miranda J., Bokde A.W., Hampel H., Ewers M. (2010). Automated detection of brain atrophy patterns based on MRI for the prediction of Alzheimer’s disease. Neuroimage.

[21] Klöppel S., Stonnington C.M., Chu C., Draganski B., Scahill R.I., Rohrer J.D., Fox N.C., Jack C.R., Ashburner J., Frackowiak R.S.J. (2008). Automatic classification of MR scans in Alzheimer’s disease. Brain.

[22] Gray K.R., Aljabar P., Heckemann R.A., Hammers A. (2013). NeuroImage Random forest-based similarity measures for multi-modal classi fi cation of Alzheimer’s disease. Neuroimage.

[23] Morra J.H., Tu Z., Apostolova L.G., Green A.E., Toga A.W., Thompson P.M. (2010). Comparison of adaboost and support vector machines for detecting Alzheimer’s disease through automated hippocampal segmentation. IEEE Trans. Med. Imaging.

[24] Neffati S., Ben Abdellafou K., Jaffel I., Taouali O., Bouzrara K. (2019). An improved machine learning technique based on downsized KPCA for Alzheimer’s disease classification. Int. J. Imaging Syst. Technol..

[25] Zhang Y., Wang S. (2015). Detection of Alzheimer’s disease by displacement field and machine learning. PeerJ.

[26] Ben Ahmed O., Benois-Pineau J., Allard M., Ben Amar C., Catheline G. (2014). Classification of Alzheimer’s disease subjects from MRI using hippocampal visual features. Multimed. Tools Appl..

[27] El-Dahshan E.S.A., Hosny T., Salem A.B.M. (2010). Hybrid intelligent techniques for MRI brain images classification. Digit. Signal Process. A Rev. J..

[28] Hinrichs C., Singh V., Mukherjee L., Xu G., Chung M.K., Johnson S.C. (2009). Spatially augmented LPboosting for AD classification with evaluations on the ADNI dataset. Neuroimage.

[29] Yue L., Gong X., Li J., Ji H., Li M., Nandi A.K. (2019). Hierarchical feature extraction for early Alzheimer’s disease diagnosis. IEEE Access.

[30] Ahmed S., Choi K.Y., Lee J.J., Kim B.C., Kwon G.R., Lee K.H., Jung H.Y. (2019). Ensembles of patch-based classifiers for diagnosis of Alzheimer diseases. IEEE Access.

[31] Chaturvedi I., Cambria E., Welsch R.E., Herrera F. (2018). Distinguishing between facts and opinions for sentiment analysis: Survey and challenges. Inf. Fusion.

[32] Zeng K., Yang Y., Xiao G., Chen Z. (2019). A very deep densely connected network for compressed sensing MRI. IEEE Access.

[33] Zhang Y.Y., Dong Z., Phillips P., Wang S.H., Ji G., Yang J., Yuan T.F., Zhang D., Wang Y., Zhou L. (2015). Ultrafast 3D ultrasound localization microscopy using a 32 × 32 matrix array. IEEE Trans. Med. Imaging.

[34] Gupta A., Ayhan M.S., Maida A.S. (2013). Natural image bases to represent neuroimaging data. 30th Int. Conf. Mach. Learn..

[35] Dou Q., Member S., Chen H., Member S., Yu L., Zhao L., Qin J. (2016). Automatic detection of cerebral microbleeds from MR images via 3D convolutional neural networks. IEEE.

[36] Suk H., Lee S., Shen D. (2017). Deep ensemble learning of sparse regression models for brain disease diagnosis. Med. Image Anal..

[37] Hong X., Lin R., Yang C., Zeng N., Cai C., Gou J., Yang J. (2019). Predicting Alzheimer’s disease using LSTM. IEEE Access.

[38] Fulton L.V., Dolezel D., Harrop J., Yan Y., Fulton C.P. (2019). Classification of Alzheimer’s disease with and without imagery using gradient boosted machines and ResNet-50. Brain Sci..

[39] Jenkins N.W., Lituiev M.S.D., Timothy P., Aboian M.P.P.M.S. (2018). A deep learning model to predict a diagnosis of Alzheimer disease by using 18 F-FDG PET of the brain. Radiology.

[40] Khan N.M., Abraham N., Hon M. (2019). Transfer learning with intelligent training data selection for prediction of Alzheimer’s disease. IEEE Access.

[41] Gorji H.T., Kaabouch N. (2019). A deep learning approach for diagnosis of mild cognitive impairment based on mri images. Brain Sci..

[42] Severyn A., Moschitti A. UNITN: Training deep convolutional neural network for twitter sentiment classification. Proceedings of the 9th International Workshop on Semantic Evaluation (SemEval 2015).

[43] Liu S., Deng W. Very deep convolutional neural network based image classification using small training sample size. Proceedings of the 2015 3rd IAPR Asian Conference on Pattern Recognition (ACPR).

[44] Kim H., Jeong Y.S. (2019). Sentiment classification using convolutional neural networks. Appl. Sci..

[45] Chincarini A., Bosco P., Calvini P., Gemme G., Esposito M., Olivieri C., Rei L., Squarcia S., Rodriguez G., Bellotti R. (2011). Local MRI analysis approach in the diagnosis of early and prodromal Alzheimer’s disease. Neuroimage.

[46] Ateeq T., Majeed M.N., Anwar S.M., Maqsood M., Rehman Z.-u., Lee J.W., Muhammad K., Wang S., Baik S.W., Mehmood I. (2018). Ensemble-classifiers-assisted detection of cerebral microbleeds in brain MRI. Comput. Electr. Eng..

[47] Yang J., Zhao Y., Chan J.C.W., Yi C. (2017). The effectiveness of data augmentation in image classification using deep learning. arXiv.

[48] Bjerrum E.J. (2015). SMILES enumeration as data augmentation for neural network modeling of molecules. arXiv.

[49] Schmidhuber J. (2015). Deep learning in neural networks: An overview. Neural Networks.

[50] Ioffe S., Szegedy C. (2015). Batch normalization: Accelerating deep network training by reducing internal covariate shift. arXiv.

[51] Wiesler S., Richard A., Schl R. Mean-Normalized Stochastic Gradient for large-scale deep learning. Proceedings of the International Conference on Acoustics, Speech and Signal Processing (ICASSP).

[52] Boyat A.K., Joshi B.K. (2015). A review paper: noise models in digital image processing. arXiv.

[53] Islam J., Zhang Y. (2018). Brain MRI analysis for Alzheimer’s disease diagnosis using an ensemble system of deep convolutional neural networks. Brain Informatics.

[54] Asl E.H., Ghazal M., Mahmoud A., Aslantas A., Shalaby A., Casanova M., Barnes G., Gimel’farb G., Keynton R., Baz A. (2018). El Alzheimer’s disease diagnostics by a 3D deeply supervised adaptable convolutional network. Front. Biosci. Landmark.

[55] Evgin G. (2019). Diagnosis of Alzheimer’s disease with Sobolev gradient- based optimization and 3D convolutional neural network. Int. J. Meth. Biomed. Eng..

[56] Farooq A., Anwar S., Awais M., Rehman S. A deep CNN based multi-class classification of Alzheimer’s disease using MRI. Proceedings of the 2017 IEEE International Conference on Imaging Systems and Techniques (IST).

